# Bayesian Estimation of Small Effects in Exercise and Sports Science

**DOI:** 10.1371/journal.pone.0147311

**Published:** 2016-04-13

**Authors:** Kerrie L. Mengersen, Christopher C. Drovandi, Christian P. Robert, David B. Pyne, Christopher J. Gore

**Affiliations:** 1 Science and Engineering Faculty, Mathematical Sciences, and Institute for Future Environments, Queensland University of Technology, Brisbane, Australia; 2 Australian Research Council Centre of Excellence in Mathematical and Statistical Frontiers in Big Data, Big Models and New Insights, Brisbane, Australia; 3 Ceremade, Universite Paris Dauphine, Paris, France; 4 Australian Institute of Sport, Canberra, Australia; 5 Research Institute for Sport and Exercise, University of Canberra, Bruce, ACT, Australia; 6 Exercise Physiology Laboratory, Flinders University of South Australia, Bedford Park, South Australia; Feng Chia University, TAIWAN

## Abstract

The aim of this paper is to provide a Bayesian formulation of the so-called magnitude-based inference approach to quantifying and interpreting effects, and in a case study example provide accurate probabilistic statements that correspond to the intended magnitude-based inferences. The model is described in the context of a published small-scale athlete study which employed a magnitude-based inference approach to compare the effect of two altitude training regimens (live high-train low (LHTL), and intermittent hypoxic exposure (IHE)) on running performance and blood measurements of elite triathletes. The posterior distributions, and corresponding point and interval estimates, for the parameters and associated effects and comparisons of interest, were estimated using Markov chain Monte Carlo simulations. The Bayesian analysis was shown to provide more direct probabilistic comparisons of treatments and able to identify small effects of interest. The approach avoided asymptotic assumptions and overcame issues such as multiple testing. Bayesian analysis of unscaled effects showed a probability of 0.96 that LHTL yields a substantially greater increase in hemoglobin mass than IHE, a 0.93 probability of a substantially greater improvement in running economy and a greater than 0.96 probability that both IHE and LHTL yield a substantially greater improvement in maximum blood lactate concentration compared to a Placebo. The conclusions are consistent with those obtained using a ‘magnitude-based inference’ approach that has been promoted in the field. The paper demonstrates that a fully Bayesian analysis is a simple and effective way of analysing small effects, providing a rich set of results that are straightforward to interpret in terms of probabilistic statements.

## Introduction

A key interest in sports science is the estimation and evaluation of small effects, such as the difference in finishing times between world-class athletes, or the impact of exercise training and/or lifestyle interventions such as dietary changes or sleep behaviors on performance [1]. While such an interest is not confined to this context [2], there are some features of sports science that make accurate and relevant estimation of small effects particularly challenging. Two such challenges are small sample sizes when dealing with international-standard, elite-level athletes and frequent small true between-individual differences in competitive performance. The issue of dealing with small sample sizes in studies has drawn comment in the fields of both medicine [3, 4] and sports science [5].

These issues have been addressed by a number of sports science researchers. For example, Batterham and Hopkins (2006) challenged the traditional method of making an inference based on a p-value derived from a hypothesis test, arguing that it is confusing, potentially misleading and unnecessarily restrictive in its inferential capability [6]. The authors suggested alternative is to focus on the confidence interval as a measure of the uncertainty of the estimated effect, and examine the proportion of this interval that overlaps pre-defined magnitudes that are clinically or mechanistically relevant. As illustration, Batterham and Hopkins identify ‘substantially positive’, ‘trivial’ and ‘substantially negative’ magnitudes, as well as more finely graded magnitudes. The authors then translate these proportions to a set of likelihood statements about the magnitude of the true effect.

Batterham and Hopkins justify their suggested approach and corresponding inferences by drawing an analogy between their method and a Bayesian construction of the problem. In particular, they claim that their approach is approximately Bayesian based on no prior assumption about the distribution of the true parameter values. This has drawn criticism by a number of authors, such as Barker and Schofield (2008) who–rightly–point out that the approach is *not* Bayesian, and that the assumed priors in an analogous Bayesian approach may indeed be informative [7]. More recently, Welsh and Knight (2014) further criticised the approach of Batterham and Hopkins and suggested that relevant statistical approaches should use either confidence intervals or a fully Bayesian analysis [8].

The aim of this paper is to provide a Bayesian formulation of the method proposed by Batterham and Hopkins (2006) and provide a range of probabilistic statements that parallel their intended magnitude-based inferences. The models described here can be expanded as needed to address other issues. For further exposition, the model is described in the context of a small-scale athlete study authored by Humberstone-Gough and co-workers [9], which employed Batterham and Hopkins’ approach to compare the effect of two altitude training regimens (live high train low, and intermittent hypoxic exposure) on running performance and blood measurements of elite triathletes.

## Methods

### General model

Both Bayesian and frequentist approaches require specification of a statistical model for the observed data, which contains a number of parameters that need to be estimated. Bayesian methods are different from frequentist approaches in that the parameters are treated as random variables. That is, they are considered as having true, but unknown, values and are thus described by a (posterior) probability distribution that reflects the uncertainty associated with how well they are known, based on the data. The posterior distribution is obtained by multiplying the likelihood, which describes the probability of observing the data given specified values of the parameters, and the prior distribution(s), which encapsulates beliefs about the probability of obtaining those parameter values independently of the data. These priors may be developed using a range of information sources including previous experiments, historical data and/or expert opinion. Alternatively, they may be so-called uninformative or vague distributions, to allow inferences to be driven by the observed data.

This study describes a simple statistical model that might be considered in the context of examining small effects in sports science and also some possible prior distributions that might be placed on the parameters of this model. Some extensions to the model are considered in a later section.

Suppose that there are *G* treatment groups. For the *g*th group (*g =* 1,…,*G*), let *n*_*g*_ denote the total number of individuals in the group, *y*_*i*(*g*)_ denote an observed effect of interest for the *i*th individual in the group (*i* = 1,…,*n*_*g*_), *y*_*g*_ denote the set of observations in the group, ${\overset{-}{y}}_{g}$ and $s_{g}^{2}$ denote respectively the sample mean and sample standard deviation of all the observed responses from the group, and *v*_*g*_ = *n*_*g*_−1 denote the degrees of freedom. For example, in the following case study, there are *G* = 3 groups (training regimens); *y*_*i(g)*_ is the difference between the post- and pre-treatment measurements for a selected response for the *i*th athlete in the *g*th training regimen, and ${\overset{-}{y}}_{g}$ is the average difference for that group.

Assume that an observation *y*_*i*(*g*)_ is Normally distributed around a group mean *μ*_*g*_, with a group-specific variance $\sigma_{g}^{2}$, i.e.:

$$y_{i\left(g\right)}\sim \text{Normal(}\mu_{g},\sigma_{g}^{2})$$ (1)

A vague prior density is adopted for the pair of parameters $\left(\mu_{g},\sigma_{g}^{2}\right)$ [10] so that:

$$p\left(\mu_{g},\sigma_{g}^{2}\right)\propto \sigma_{g}^{-2}$$ (2)

(where *∝* denotes proportional to). Based on [1] and [2], the posterior conditional distributions for *μ*_*g*_ and $\sigma_{g}^{2}$ are given by

$$\mu_{g}|\sigma_{g}^{2},y_{g}\sim \text{N(}{\bar{y}}_{g},\frac{\sigma_{g}^{2}}{n_{g}})$$ (3)

$$\sigma_{g}^{2}|y_{g}\sim \text{Inverse}\chi^{2}(\upsilon_{g},s_{g}^{2}).$$ (4)

The marginal posterior distribution for *μ*_*g*_ can be shown to have a *t* distribution on *v*_*g*_ degrees of freedom: [10]

$$\mu_{g}|y_{g}\sim t_{g}\text{(}{\bar{y}}_{g},\frac{s_{g}^{2}}{n_{g}})$$ (5)

so that

$$(\mu_{g}-{\bar{y}}_{g})/(s_{g}^{2}/\sqrt{n_{g})}|y_{g}\sim t_{\upsilon g}$$ (6)

### Relationship with frequentist results

The marginal posterior distributions for $\sigma_{g}^{2}$ and *μ*_*g*_, based on the data, are given by Eqs (4) and (5), respectively. Because of the choice of the vague prior (Eq (2)), these distributions can be shown to be closely related to analogous distributions for the (appropriately scaled) sufficient statistics, given *μ*_*g*_ and $\sigma_{g}^{2}$, based on frequentist sampling theory: [10]

$$\upsilon_{g}s_{g}^{2}|\sigma_{g}^{2}\sim \chi_{\upsilon g}^{2}$$ (7)

$$({\bar{y}}_{g}-\mu_{g})/(s_{g}^{2}/\sqrt{n_{g})}\sim t_{\upsilon g}.$$ (8)

### Estimation of values of interest

A range of posterior estimates (conditional on the available data) arising from the model may be of interest, including:

1. the mean and standard deviation for each group (e.g., each training regimen in the study), given by *μ*_*g*_ and $\sigma_{g}^{2}$, respectively
2. the difference between the group means: *δ*_*kl*_ = *μ*_*k*_−*μ*_*l*_ and the associated standard deviation of this difference, *σ*_*kl*_
3. a (1−*α*)% credible interval (CI) for a measure of interest, *θ*, say, such that there is a posterior probability (1−*α*) that *θ* lies in this interval (e.g., *θ* could be the mean of group 2, i.e., *θ = μ*_2_, and a 95% CI of (3.1, 4.2), for instance, indicates that the probability that *μ*_2_ is between 3.1 and 4.2, given the data, is 0.95), which is a much more direct statement than is possible under a frequentist approach
4. Cohen’s *d* [11] for the difference between two groups, given by *d*_*kl*_ = *δ*_*kl*_/*σ*_*kl*_ when comparing groups *k* and *l*, *k* ≠ *l*
5. the probability that Cohen’s *d* exceeds a specified threshold such as a ‘smallest worthwhile change’ (*SWC*,[6]), given by Pr(*d*_*kl*_ > SWC) or Pr(*d*_*kl*_ < −SWC), depending on whether *d*_*kl*_ is positive or negative, respectively
6. the predicted outcome of each individual under each training regimen, regardless of whether or not they have participated in that training, obtained from Eq (1), with an estimate of the corresponding uncertainty of this prediction
7. the ranks of each individual under each training regimen, with corresponding uncertainty in these orderings.

Given the data *y*_*g*_ for each group (and hence the sufficient statistics ${\overset{-}{y}}_{g}$ and $s_{g}^{2}$), it is straightforward to use Eqs (4) and (5) to compute posterior estimates *μ*_*g*_ and $\sigma_{g}^{2}$, and other probabilities of interest. An alternative, simple approach is to simulate values of interest using Eqs (3) and (4) iteratively, employing a form of Markov chain Monte Carlo (MCMC) [12]. A more technical explanation of this approach including the Gibbs sampling techniques is provided by Geman and Geman [13]. At each iteration, a value of $\sigma_{g}^{2}$ is simulated from Eq (4) and then a value of *μ*_*g*_ given that value of $\sigma_{g}^{2}$ is simulated from Eq (3). This process is repeated a large number of times. The simulated values can be used to compute other measures (e.g. exp(*μ*_1_−*μ*_2_) if this is of interest), indicators I(*μ*_1_ > *c*) or I(*μ*_1_ > *μ*_2_) and so on. Then E(exp(*μ*_1_−*μ*_2_)), Pr(*μ*_1_ > *c*) and Pr(*μ*_1_ > *μ*_2_) can be estimated (where E denotes expectation) as the respective averages of these values over all of the iterations. Similarly, at each iteration, the simulated parameter values can be input into Eq (1) to obtain predicted values of *y* for each individual under each regimen, and the individuals can be ranked with respect to their predicted outcome. The posterior distributions for individual predicted outcomes, and the probability distribution for the ranks, are computed from the respective values obtained from the set of iterations.

The Cohen’s *d* is a standardized effect size estimate, calculated as the difference between two means divided by the corresponding standard deviation. While there are many effect size estimators, Cohen’s *d* is one of the most common since it is appropriate for comparing between the means of distinctly different group and it has appealing statistical properties; for example it has a well known distribution and is maximum likelihood estimator [14].

### Model extensions

The model described above can be easily extended in a range of ways. Three such extensions are considered here. The first extension is that other prior distributions can be considered instead of Eq (2) above. For example, another common approach is to assign a normal distribution for the group means,

$$\mu_{g}\sim \text{Normal(M,V)}$$ (9)

and a Uniform distribution for the standard deviations,

$$\sigma_{g}\sim \text{Uniform(0,R),}$$ (10)

where M and V denote the mean and variance of the normal distribution, respectively, and R is the upper bound of the uniform distribution. Alternatives to the uniform are the half-normal or half-Cauchy. If the sample sizes within groups are small and little is known *a priori* about the comparative variability of measurements within and between the groups, then $\sigma_{g}^{2}$ can be imprecisely estimated; to avoid this, the individual variances be replaced by a common variance, *σ*^2^ say.

There are many ways of setting the values of M, V and R. For example, if there is no prior information about these values and if the groups are considered to be independent, this can be reflected by specifying very large values of V and R, relative to the data. This means that the priors in Eqs (9) and (10) will have negligible weight in the posterior estimates of the group means *μ*_*g*_ and variances $\sigma_{g}^{2}$. If V is sufficiently large, the value of M will not matter, so it is commonly set to 0 in this case. Alternatively, the groups can be perceived as having their own characteristics (described by *μ*_*g*_ and $\sigma_{g}^{2}$) but also being part of a larger population with an overall mean M and variance V. This random effects model is very common as it helps to accommodate outliers and improve estimation of small groups. Another alternative is to use other information to set the values of M, V and R. This information can include results of previous similar experiments, published estimates, expert opinion, and so on. Depending on the problem and the available information, different values of M, V and R can be defined for the different groups. The Bayesian framework can be very helpful in providing a mechanism for combining these sources of information in a formal manner.

The second extension is that the model described in Eq (1) can be expanded to include explanatory variables that can help to improve the explanation or prediction of the response. This is the model that is adopted in the case study described below, where the explanatory variables comprise the group label and a covariate reflecting training-induced changes. For this purpose, Eq (1) is extended as follows:

$$y_{i}=x_{i}^{\prime }\beta +\varepsilon$$ (11)

where the explanatory variables and their regression coefficients are denoted by *x* and *β*, respectively, and *ε*_*i*_ describes the residual between the observation *y*_*i*_ and its predicted value $x_{i}^{'}\beta$. Note that the superscript *′* denotes the transpose. It is common to assume that *∈*_*i*_~Normal(0,*σ*^2^). Normally distributed priors are placed on the parameters in this regression model:

$$\beta \sim {\text{Normal}}_{k}\text{(}b_{0},{\text{B}}_{0}^{-1});\sigma^{2}\sim \text{Gamma(}c_{0}/2,\;d_{0}/2)$$ (12)

where *k* represents the number of parameters, Normal_*k*_ indicates a *k*-dimensional Gaussian distribution and Gamma indicates a Gamma distribution described by shape and scale parameters, in this case given by constants *c*_0_ and *d*_0_.

An uninformative prior specification can be defined for *β* by setting zero values for the mean vector *b*_0_ and precision matrix *B*_0_. Similarly, negligible prior information about the magnitude of the residuals is reflected by setting small values for *c*_0_ and *d*_0_ in the distribution for *σ*^2^ [15].

An alternative, popular formulation is to use Zellner’s *g*-prior, whereby the variance of the prior for *β* is defined in terms of the variance for the data. More explicitly, b is specified as a multivariate normal distribution with a covariance matrix that is proportional to the inverse Fisher information matrix for *β*, given by *g*(*x*^*T*^*x*)^−1^. This is an elegant way of specifying the ‘information’ contained in the prior, relative to that contained in the data: the value of *g* is analogous to the ‘equivalent number of observations’ that is contributed to the analysis by the prior [16, 17].

The third extension is the choice of the response *y*. This depends on the aim of the analysis, biological and other contextual knowledge of the problem, and the available data. The residuals are assumed to have a normal distribution with a mean of zero, and normally distributed priors can be defined as the difference between an individual’s post-training and pre-training measurements, the difference of the logarithms of these measurements, the relative difference between the pairs of measurements (i.e. (post-pre)/pre) or some other context-relevant transformation.

### Case Study

The Bayesian approach described above was applied to a study by Humberstone-Gough *et al*. [9] who used a two-period (pre-post) repeated measures design to compare the effects of three training regimens ‘Live High Train Low’ altitude training (LHTL), ‘Intermittent Hypoxic Exposure’ (IHE) and ‘Placebo’ on running performance and blood characteristics. The study comprised eight subjects (elite male triathletes) in each regimen, and had one dropout in the LHTL group. Although ten running and blood variables were considered in the original study; three variables with the most complete data are selected here for illustration: hemoglobin mass (Hbmass, units of grams), submaximal running economy (RunEcon, units of L O_2_.min^-1^) and maximum blood lactate concentration (La-max, units of mmol/L). The authors also employed a covariate reflecting training-induced changes, namely the percent change in weekly training load from pre- to during-camp for each individual athlete. The data used for the analyses are shown in S1 Table (data extracted from original study of Humberstone-Gough et al (2013 and provided by co-author Gore).

Casting this study in terms of the models described above, there are *G = 3* groups denoting the training regimens (Placebo by *g* = 1; IHE by *g* = 2; LHTL by *g* = 3). Letting pre_*i*_ and post_*i*_ denote respectively the pre- and post-treatment measurements for the *i*th individual, an (unscaled) effect of interest, *y*_*i*_, was defined in terms of the difference between the pairs of measurements:

$$y_{i}={\text{post}}_{i}-{\text{pre}}_{i}.$$ (13)

A log transformation was adopted in the original analysis by Humberstone-Gough *et al*. [9] but was not performed in the analysis described below, as there was insufficient information in the observed data to strongly motivate a transformation of the measurements, particularly after adjusting for the covariate reported by Humberstone-Gough *et al*. (comparative summary plots not shown). However, it is acknowledged that this decision was based purely on the available data and there may be compelling biological or experimental reasons for choosing the log (or other) scale; for example, under this transformation covariates can be considered to have multiplicative rather than additive effects on the original response. On the one hand, retaining the original scale allows for more direct interpretation of the results. On the other, if the underlying assumptions are not met, the inferences based on the results must be treated with caution. In this study, the premise was adopted of not transforming unless there is a compelling domain-specific or statistical reason to do so. Hence the decision was made not to take a log transformation of the data as other authors have suggested–a statistical decision–and to consider a relative change in performance as well as an absolute difference–a domain-based decision since this measure is of interest to sports scientists. A similar issue arises about the inclusion of covariates in a small sample analysis. In this case, the associated regression parameters may be estimated with substantial uncertainty and the usual model comparison methods are often inadequate in determining any associated improvement in model fit. Again, the decision may be more domain-based than statistical. In the study considered here, results were reported with and without a covariate that was considered to be important for sports scientists, and a deliberate decision was made to avoid formal model comparison. These issues of data transformation and model comparison for small samples merit further research.

Here we consider instead an analogous scaled effect defined in terms of the relative difference between the pairs of measurements:

$$y_{i}\;=\;{\text{(post}}_{i}-{\text{pre}}_{i})/{\text{pre}}_{i}.$$ (14)

For both the unscaled response given by Eq (13) and the relative response given by Eq (14), the list of posterior estimates of interest were:

- the differences between the two experimental training regimens (IHE, LHTL) and the Placebo group, given by *δ*_*12*_ and *δ*_*13*_, respectively, and the difference between the two training regimens IHE and LHTL, given by *δ*_*23*_;
- Cohen’s *d* for each of the two experimental regimens compared with the Placebo regimen, given by *d*_*12*_ = *δ*_*12*_/*σ*_*12*_ for IHE and *d*_*13*_ = *δ*_*3*_/*σ*_*13*_ for LHTL;
- Cohen’s *d* for the standardized difference between LHTL versus IHE, given by *d*_*23*_ = *δ*_*23*_/*σ*_*23*_;
- the probabilities that the standardized difference between the IHE training regimen and the Placebo exceed the ‘smallest worthwhile change’ (*SWC*, specified as a standardised change of *0*.*2* based on previous recommendations [18]), denoted by *SWCU*_*12*_ = Pr(*d*_*12*_ > 0.2) and *SWCL*_*12*_ = Pr(*d*_*12*_ < -0.2);
- analogous probabilistic comparisons with the *SWC* for the difference between the LHTL training regimen and the Placebo, and the LHTL and IHE training regimens,
- the posterior distributions of the expected outcome E(*y*_*ij*_) = *β*_0_ + *β*_1_*X* + *β*_2_*I*_*j* = 1_ + *β*_3_*I*_*j* = 2_ for the *i*th individual under the *j*th training regimen (where *I*_*j = 1*_ = 1 if the treatment is IHE and = 0 otherwise, and *I*_*j = 2*_ = 1 if the treatment is LHTL and = 0 otherwise); the expected outcome, obtained by substituting the simulated parameter values (*β*_0_, *β*_1_, *β*_2_, *β*_3_) into this equation at each MCMC simulation,
- the analogous posterior predicted outcome for each individual under each training regimen, which allows for within-subject variation around the expected outcome, i.e., $y_{ij}^{\text{pred}}=y_{ij}^{\text{pred}}+e_{ij},\;\;e_{ij}\sim \text{N}\left(0,\sigma^{2}\right)$, which is obtained in the same manner as above,
- the ranks of the individuals based on their expected and predicted outcomes under each of the treatment regimens; again, this is a probability distribution, reflecting the fact that rankings may change depending on the precision of the estimated treatment effects and within-subject variation.

Note that although the denominator of the Cohen’s *d*_*kl*_ values can be calculated using the traditional equation, i.e., *σ*_*kl*_ = √Var(*δ*_*l*_-*δ*_*k*_) = √((*v*_*l*_Var(*δ*_*l*_)+*v*_*k*_Var(*d*_*k*_))/(*v*_*l*_+*v*_*k*_)), this can also be directly calculated using the simulated values of *d*_*kl*_ obtained from the MCMC iterations, i.e., *σ*_*kl*_ = √Var(*d*_*kl*_).

Based on exploratory plots of the relationships between the observed pre- and post-training values of Hbmass, RunEcon and La-max among the three groups, and with the covariate, two analyses of the data were undertaken. In the first analysis, the covariate was excluded and the model was fit using Eqs (3) and (4). In the second analysis, the covariate was included given previous work showing that training load can influence the hemopoietic response [19] and the model was fit using Eq (11). The models were implemented using the statistical software R, with packages BRugs and R2WinBugs, which call WinBUGS [15, 20, 21], and MCMCregress in the MCMCpack library [22]. Estimates were based on 150,000 MCMC iterations, after discarding an initial burn-in of 50,000 iterations. For comparability with Humberstone-Gough *et al* [9], the results of the second analysis are reported below. The R code for this model is presented as a text file in S1 Text.

As described above, the primary analyses for the case study utilized an uninformative prior specification for β in Eq (12), which was obtained by setting the values of the prior mean vector *b*_0_ and prior precision matrix B_0_ to zero. The impact of informative priors was evaluated by considering a range of non-zero values for these terms, with Hbmass as the response measure. The values were motivated by the results of a recent meta-analysis of training regimens on Hbmass [23], which reported a mean response of 1.08% increase in Hbmass per 100 hours of LHTL training. Based on the study of Humberstone-Gough with 240 hours of exposure, the prior expectation is thus that the mean increases for the LHTL and IHE groups would be 2.6% and 0% respectively. The latter figure is also supported by a report that 3 h/day at 4000–5,500 m was inadequate to increase Hbmass at all [24]. This literature also provides a prior expectation of 0% increase in Hbmass of the Placebo group. The 2013 meta-analysis [23] also provided an estimate of 2.2% for the within-subject standard deviation of Hbmass.

## Results

**Fig 1:**
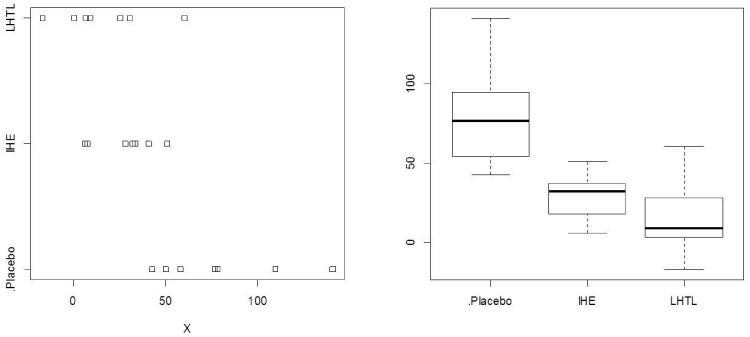
Exploratory analyses comprising stripcharts (left) and boxplots (right) for the covariate X in the three training regimens (Placebo, Intermittent Hypoxic Exposure (IHE), Live High Train Low (LHTL)), where X is a measure of the percent change in training load for each of the 23 individuals in the study. (See text for details.).

The distribution of the covariate X (representing the % change in weekly training load from pre- to post-camp) within and among the three training regimens (Placebo, IHE, LHTL) is displayed in Fig 1. The plots show that there is non-negligible variation between individuals within a regimen with respect to this variable and substantive differences between the regimens. It is clear that adjustment needs to be made for X before evaluating the comparative impact of the three regimens. This is accommodated in the regression model described in Eq (11).

**Fig 2:**
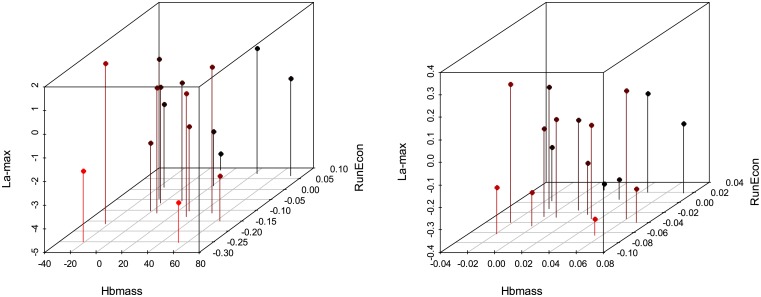
Three-dimensional scatterplot of the three measurements, Hemoglobin Mass (Hbmass), Running Economy (RunEcon) and maximum blood lactate concentration (La-max), unscaled data (left) and scaled data (right). Unscaled data are calculated as post_*i*_−pre, and scaled data are calculated as (post_*i*_−pre_*i*_) / pre_*i*_.

**Fig 3:**
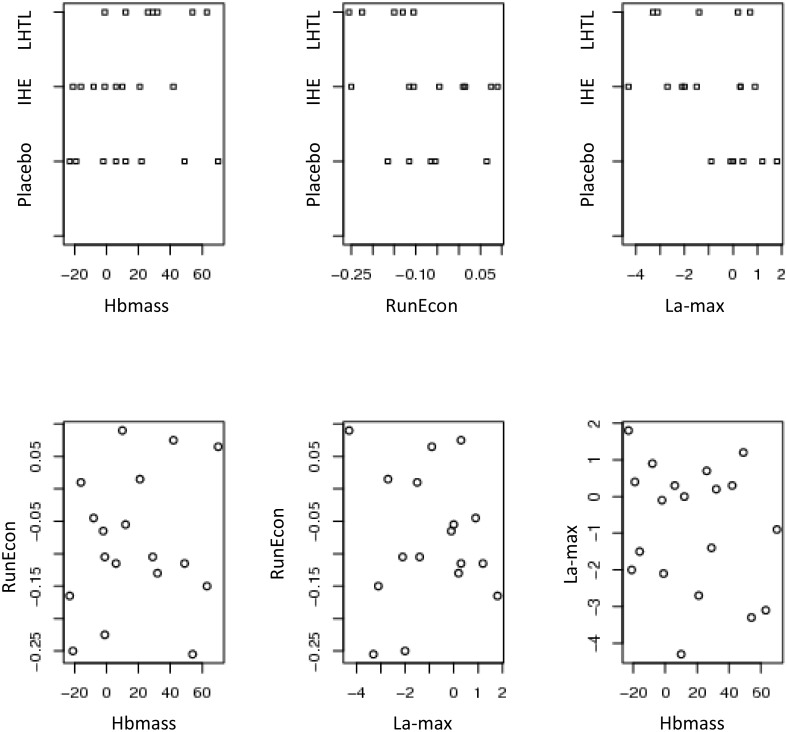
Two-dimensional scatterplots of the three measurements of Hbmass, RunEcon and La-max, under three regimes Placebo, Intermittent Hypoxic Exposure (IHE) and Live High Train Low (LHTL), unscaled data.

**Fig 4:**
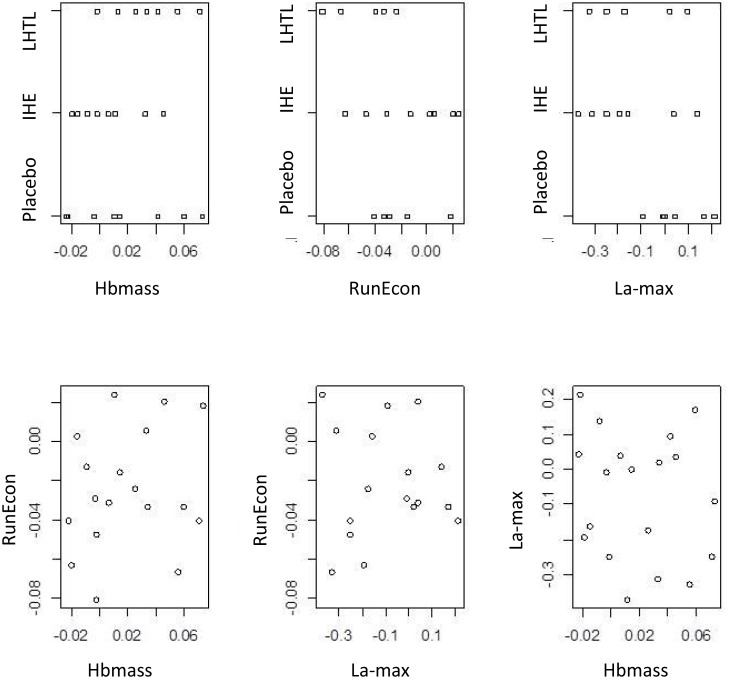
Two-dimensional scatterplots of the three measurements of Hbmass, RunEcon and La-max, under three regimes Placebo, Intermittent Hypoxic Exposure (IHE) and Live High Train Low (LHTL), scaled data.

Scatterplots of the unscaled differences given by Eq (13) and scaled differences given by Eq (14) are presented in Figs 2–4. Based on these plots, there is no clear visual association between the three measurements under consideration in this case study (Hbmass, RunEcon and La-max), or between these measurements and the covariate.

**Fig 5:**
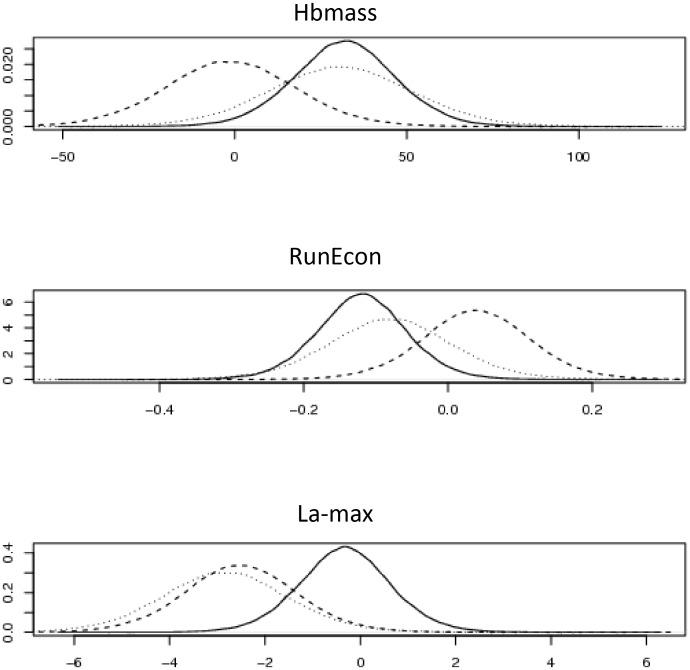
Posterior densities of the three measurements, Haemoglobin Mass, Running Economy and Running Maximum Lactate, comparing Live High Train Low (LHTL) vs Intermittent Hypoxic Exposure (IHE) (solid line), LHTL vs Placebo (dotted line) and IHE vs Placebo (dashed line), unscaled data.

Plots of the posterior distributions of the differences between the training regimens, IHE vs Placebo, LHTL vs Placebo, LHTL vs IHE, given by *δ*_*12*_, *δ*_*13*_ and *δ*_*23*_, respectively, are shown in Fig 5. Corresponding posterior estimates of the effects (mean, s.d., 95% and 90% credible intervals) are given in Table 1.

**Table 1:** Posterior estimates based on unscaled data.

**Hbmass**				
*Posterior parameter estimates (units of grams)*				
Effect	Mean	s.d.	95% CI	90% CI
X	0.25	0.25	-0.25, 0.74	-0.17, 0.66
IHE	-1.4	19.8	-40.5, 37.8	-33.8, 30.9
LHTL	30.7	21.7	-12.4, 73.4	-4.9, 66.2
LHTL-IHE	32.0	15.3	1.9, 62.2	7.04, 57.2
*Cohen’s d*				
Effect	Mean	s.d.	95% CI	90% CI
IHE	-0.07	1.0	-2.1, 1.9	-1.7. 1.6
LHTL	1.4	1.0	-0.57, 3.4	-0.23, 3.0
LHTL-IHE	2.1	1.0	0.12, 4.1	0.46, 3.7
*Prob*. *Cohen’s d <> 0*.*2*				
Parameter	Prob. *d*<-0.2	Prob. *d*>0.2		
IHE	0.45	0.39		
LHTL	0.052	0.89		
LHTL-IHE	0.013	0.97		
**RunEcon**				
*Posterior parameter estimates (unit of L/min)*				
Effect	Mean	s.d.	95% CI	90% CI
X	0.00045	0.0010	-0.0016, 0.0025	-0.0012, 0.0021
IHE	0.039	0.079	-0.12, 0.20	-0.09, 0.17
LHTL	-0.080	0.090	-0.26, 0.097	-0.23, 0.065
LHTL-IHE	-0.12	0.064	-0.25, 0.0094	-0.22, -0.014
*Cohen’s d*				
Effect	Mean	s.d.	95% CI	90% CI
IHE	0.50	1.0	-1.5, 2.5	-1.1, 2.1
LHTL	-0.89	1.0	-2.9, 1.1	-2.5, 0.73
LHTL-IHE	-1.85	1.0	-3.8, 0.15	-3.5, -0.22
*Prob*. *Cohen’s d <> 0*.*2*				
Parameter	Prob. *d*<-0.2	Prob. *d*>0.2		
IHE	0.23	0.62		
LHTL	0.77	0.13		
LHTL-IHE	0.95	0.023		
**La-max**				
*Posterior parameter estimates (units of mmol/L)*				
Effect	Mean	s.d.	95% CI	90% CI
X	-0.018	0.015	-0.050, 0.013	-0.044, 0.0076
IHE	-2.5	1.3	-5.0, -0.06	-4.6, -0.50
LHTL	-2.8	1.3	-5.6, -0.10	-5.10-, -0.59
LHTL-IHE	-0.32	1.0	-2.3, 1.66	-1.2, 1.3
*Cohen’s d*				
Effect	Mean	s.d.	95% CI	90% CI
IHE	-2.0	1.0	-4.0, -0.054	-3.7, -0.40
LHTL	-2.1	1.0	-4.0, -0.070	-3.7, -0.48
LHTL-IHE	-0.32	1.0	-2.3, 1.7	-2.0, 1.3
*Prob*. *Cohen’s d <> 0*.*2*				
Parameter	Prob. *d*<-0.2	Prob. *d*>0.2		
IHE	0.97	0.015		
LHTL	0.98	0.015		
HTL-IHE	0.55	0.29		

From Fig 5 and Table 1, it can be seen that for Hbmass and RunEcon, although there is a slight detrimental effect of IHE and a slight beneficial effect of LHTL compared with the Placebo, these are not substantive: a difference of 0 is reasonably well supported by the posterior distributions. However, this slight differential in response results between IHE and LTHL: a difference of 0 appears to have less support in the posterior densities; the 90% credible interval does not include 0 and the posterior probability that Cohen’s *d* exceeds the SWC is 0.96 and 0.93 for Hbmass and RunEcon respectively. These outcomes strongly indicate that LHTL is substantively better than IHE for both of these outcome measures.

In contrast, for La-max, both IHE and LHTL show a substantive beneficial effect compared with the Placebo, with the corresponding 95% (and hence 90%) credible intervals excluding 0 and a probability of 0.97 that Cohen’s *d* exceeds the SWC. As a consequence, the difference between LHTL and IHE is thus attenuated for this outcome measure.

**Fig 6:**
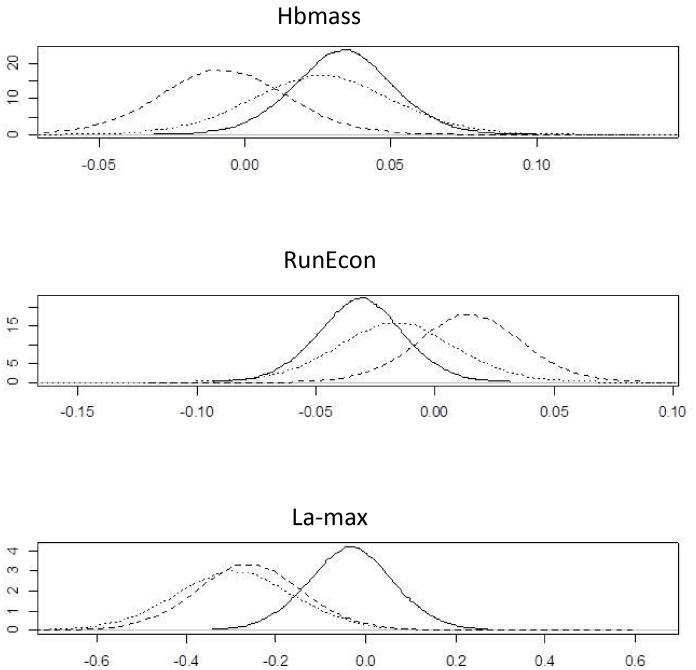
Posterior densities of the three measurements, Haemoglobin Mass, Running Economy and Running Maximum Lactate, comparing Live High Train Low (LHTL) vs Intermittent Hypoxic Exposure (IHE) (solid line), LHTL vs Placebo (dotted line) and IHE vs Placebo (dashed line), scaled data.

Posterior estimates of parameters of interest for the scaled (relative) measures are shown in Fig 6 and Table 2. The figures and table confirm the above results. Similar to the unscaled effects, there is no clear visual association between two of the measurements under consideration in this case study (Hbmass and RunEcon), or between these measurements and the covariate of change in weekly training load. However, there is a clear difference in the values of the covariate among individuals in the Placebo group compared with the two training regimens (LHTL and IHE). The two training regimens both appear to substantively improve La-max, even after accounting for training-induced changes in the individual athletes. The direct probabilistic comparisons with the SWC provide more complete information about these treatments based on these data.

**Table 2:** Posterior estimates based on scaled data.

**Hbmass**				
*Posterior parameter estimates (units of percent / 100)*				
Effect	Mean	s.d.	95% CI	90% CI
X	0.00020	0.00029	-0.00038, 0.00077	-0.00028, 0.00067
IHE	-0.0075	0.023	-0.053, 0.038	-0.045, 0.030
LHTL	0.026	0.025	-0.023, 0.076	-0.015, 0.068
LHTL-IHE	0.034	0.018	-0.0011, 0.069	0.0050, 0.063
*Cohen’s d*				
Effect	Mean	s.d.	95% CI	90% CI
IHE	-0.33	1.0	-2.3, 1.7	-1.2, 1.3
LHTL	1.1	1.0	-0.93, 3.0	-0.60, 2.7
LHTL-IHE	1.9	1.0	-0.059, 3.9	0.28, 3.6
*Prob*. *Cohen’s d <> 0*.*2*				
Parameter	Prob. *d*<-0.2	Prob. *d*>0.2		
IHE	0.55	0.29		
LHTL	0.10	0.81		
LHTL-IHE	0.019	0.96		
**RunEcon**				
*Posterior parameter estimates (units of percent / 100)*				
Effect	Mean	s.d.	95% CI	90% CI
X	0.00023	0.00031	-0.00038, 0.00083	-0.00027, 0.00072
IHE	0.015	0.023	-0.032, 0.061	-0.024, 0.053
LHTL	-0.016	0.027	-0.069, 0.037	-0.060, 0.027
LHTL-IHE	-0.031	0.019	-0.069, 0.0071	-0.062, 0.00016
*Cohen’s d*				
Effect	Mean	s.d.	95% CI	90% CI
IHE	0.63	1.00	-1.4, 2.6	-1.0, 2.3
LHTL	-0.61	1.00	-2.6, 1.4	-2.2, 1.0
LHTL-IHE	-1.62	1.00	-3.6, 0.37	-3.3, 0.0085
*Prob*. *Cohen’s d <> 0*.*2*				
Parameter	Prob. *d*<-0.2	Prob. *d*>0.2		
IHE	0.19	0.68		
LHTL	0.67	0.20		
LHTL-IHE	0.93	0.035		
**La-max**				
*Posterior parameter estimates (units of percent / 100)*				
Effect	Mean	s.d.	95% CI	90% CI
X	-0.0019	0.0016	-0.0051, 0.0013	-0.0045, 0.00072
IHE	-0.26	0.13	-0.51, -0.0094	-0.47, -0.054
LHTL	-0.29	0.14	-0.58, -0.014	-0.52, -0.065
LHTL-IHE	-0.034	0.10	-0.24, 0.17	-0.20, 0.13
*Cohen’s d*				
Effect	Mean	s.d.	95% CI	90% CI
IHE	-2.6	1.0	-4.0, -0.074	-3.7, -0.43
LHTL	-2.1	1.0	-4.1, -0.10	-3.7, -0.46
LHTL-IHE	-0.33	1.0	-2.3, 1.7	-2.0, 1.3
*Prob*. *Cohen’s d <> 0*.*2*				
Parameter	Prob. *d*<-0.2	Prob. *d*>0.2		
IHE	0.97	0.014		
LHTL	0.97	0.014		
LHTL-IHE	0.56	0.29		

**Fig 7:**
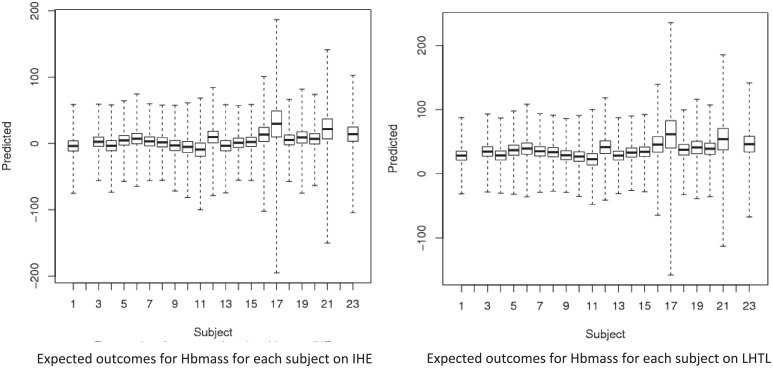
Boxplots of the posterior expected outcomes for Hbmass for each individual in the study, under each of the two training regimens Intermittent Hypoxic Exposure (left) and Live High Train Low (right).

The posterior expected outcome of Hbmass for each individual under each regimen is illustrated in Fig 7, for the unscaled data. The boxplots indicate the distribution of possible outcomes, with the box corresponding to the middle 50% of values and the limits of the bars corresponding to the minimum and maximum values. The corresponding expected rank and associated interquartile range for the 23 individuals are reported in Table 3. It is noted that the predictions and ranks are substantively driven by the covariate values in this model, with comparatively much less influence from the effect of the training regimens. Hence Table 3 displays only a selection of results.

**Table 3:** Expected rank and associated interquartile range for the 23 individuals in the study.

ID	Hbmass		RunEcon		La-max	
	Mean	IQR	Mean	IQR	Mean	IQR
1	3	3–19	3	3–19	19	3–19
2	NA	NA	NA	NA	NA	NA
3	10	10–12	10	10–12	12	10–12
4	5	5–17	5	5–17	17	5–17
5	12	10–12	12	10–12	10	10–12
6	15	7–15	15	7–15	7	7–15
7	11	11–11	11	11–11	11	11–11
8	8	8–14	8	8–14	14	8–14
9	6	6–16	6	6–16	16	6–16
10	2	2–20	2	2–20	20	2–20
11	1	1–21	1	1–21	21	1–21
12	17	5–17	17	5–17	5	5–17
13	4	4–18	4	4–18	18	4–18
14	7	7–15	7	7–15	15	7–15
15	9	9–13	9	9–13	13	9–13
16	18	4–18	18	4–18	4	4–18
17	21	1–21	21	1–21	1	1–21
18	13	9–13	13	9–13	9	9–13
19	16	6–16	16	6–16	6	6–16
20	14	8–14	14	8–14	8	8–14
21	20	2–20	20	2–20	2	2–20
22	NA	NA	NA	NA	NA	NA
23	19	3–19	19	3–19	3	3–19

A comparison of two of the primary outcome measures Hbmass and RunEcon based on the Bayesian and magnitude-based inference approach is presented in Table 4. Note that the two sets of results differ slightly not only because of differences in analytic method, but also because of differences in modelling. For example, the magnitude-based inferences are based on a log-transformed response forecast to a covariate value (a 44% increase in weekly training load), with covariate adjustment undertaken within each treatment group; in contrast, the Bayesian inferences are based on the unadjusted and relative responses forecast to the mean covariate value and adjustment is undertaken using all of the data for reasons of small sample size. Furthermore, as described above, the method of computation of the denominator of the standardized values is not based on asymptotics in the Bayesian analysis, which makes a difference for small samples. Notwithstanding these differences, the overall conclusions are similar for the two sets of analyses. For Hbmass, the Bayesian analysis indicated a substantially higher increase for LHTL with both unscaled and scaled data, with magnitude-based analysis indicating possibly higher for LHTL with unscaled data, and likely higher with scaled data. Similarly for RunEcon the outcomes were similar between the analytical approaches—the Bayesian analysis indicated a substantial improvement (lower oxygen cost) with both unscaled and scaled data, while magnitude-based analysis indicated possibly lower oxygen cost in both cases.

**Table 4:** Analysis of pre- to post-training measurements for LHTL vs IHE–outcomes for Bayesian and Magnitude-based Inferences for both unscaled and scaled data. SD = standard deviation, CL = confidence limits, CI = credible interval.

Analysis	Measure	Hemoglobin Mass	Running Economy
		(g)	(L.min^-1^)
Bayesian Unscaled	Mean ± SD	21 ± 17	-0.17 ± 0.052
	90% CI	-6, 48	-0.25, -0.08
	Cohen’s d; 90% CI	1.26; -0.37, 2.90	-3.20; -4.84, -1.57
	Probability |d|>0.2	0.931	0.998
	Qualitative inference	Higher	Lower
Magnitude-based Inference	Mean; 90% CL	36; -5, 78	-0.13; -0.22, 0.04
	Cohen’s d; 90% CL	0.18; -0.02, 0.39	-0.20; -0.34, -0.07
	Qualitative inference	Possibly Higher	Possibly Lower
Bayesian Scaled	Mean ± SD (% / 100)	0.023 ± 0.019	-0.042 ± 0.017
	90% CI	-0.008, 0.054	-0.069, -0.015
	Cohen’s d; 90% CI	1.21; -0.42, 2.85	-2.51; -4.14, -0.88
	Probabilty |d|>0.2	0.926	0.993
	Qualitative inference	Higher	Lower
Magnitude-based Inference	Smallest worthwhile difference (% / 100)	0.016	0.019
	Difference ± SD	0.047 ± 0.035	-0.028 ± 0.044
	Cohen’s d; 90% CL	0.20; 0.05, 0.35	-0.14; -0.34, 0.07
	Qualitative inference	Likely Higher	Possibly Lower

**Fig 8:**
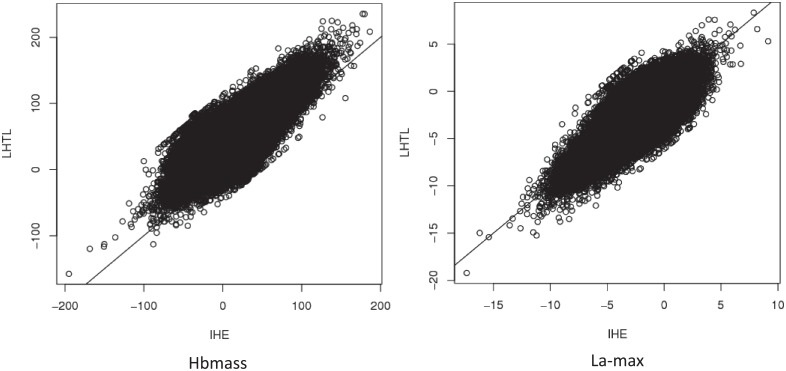
Comparison of the posterior distributions of the expected measurements of Hbmass (left) and La-max (right) under each of the training regimens Intermittent Hypoxic Exposure (IHE) and Live High Train Low (LHTL), unscaled data.

Comparison of the expected values of Hbmass and La-max under each of the training regimens is further illustrated in Fig 8. The diagonal line indicates no treatment effect. The cloud of points represents the values obtained from the MCMC simulations in the Bayesian analysis. Displacement of the cloud from the line indicates that that there is an expected improvement or decline in the outcome measure associated with the respective treatment, and the range of values for which this is anticipated to take effect.

The alternative priors that were motivated by the available external information are shown in Table 5. The consequent changes in the parameter values arising from the incorporation of these priors in the model are also shown in this table. It is clear that although the parameter estimates change slightly, the inferences reported above are generally robust to relatively small changes in the priors. However, the posterior estimates start to differ in a natural manner when the priors become more informative with respect to either the mean or variance. It is also noted that, reassuringly, the original (vague prior) setting yielded a posterior estimate of a relative increase of 2.6% in Hemoglobin mass under the LHTL regimen, which is equivalent to the anticipated value based on the (independent) prior information.

**Table 5:** Configurations of hyperparameter values for informative priors in the Bayesian model [Eqs (11 and 12)]. Here *b*_*0*_ and B_0_ denote respectively the prior mean vector and precision matrix for the regression coefficients, and *c*_*0*_*/2* and *d*_*0*_*/2* denote respectively the shape parameter and scale parameter for the inverse Gamma prior on σ^2^ (the variance of the disturbances). These latter two parameters can be respectively interpreted as indicating the amount of information, and the sum of squared errors, from c_0_ pseudo-observations, for the inverse Gamma prior on *σ*^*2*^ (the variance of the residuals) [16]. Note that (a) depicts the baseline uninformative priors used in the primary analyses, whereas (b) to (h) illustrate seven alternate priors.

Setting	(a)	(b)	(c)	(d)	(e)	(f)	(g)	(h)
*b*_*0*_	(0,0,0,0)	(0,0,0,2.6)	(0,0,0,2.6)	(0,0,0,2.6)	(0,0,0,0)	(0,0,0,0)	(0,0,0,0)	(0,0,0,2.6)
diag(B_0_)	(0,0,0,0)	(0,0,.2,.2)	(5,5,5,5)	(0,0,5,5)	(0,0,0,0)	(0,0,0,0)	(0,0,5,5)	(0,0,0,0)
*c*_*0*_	0.0001	0.0001	0.0001	0.0001	20	20	20	20
*d*_*0*_	0.0001	0.0001	0.0001	0.0001	100	5	100	100
Int.	0.00047	0.0044	-0.0027	-0.0028	0.011	0.0060	-0.64	0.0060
	(0.026)	(0.026)	(0.026)	(0.026)	(1.4)	(0.30)	(0.28)	(0.30)
X	0.00020	0.00020	0.00026	0.00026	0.00018	0.00019	0.0062	0.00019
	(0.00029)	(0.00029)	(0.00029)	(0.00029)	(0.015)	(0.0034)	(0.0033)	(0.0034)
IHE	-0.0075	-0.0073	-0.0021	-0.0021	-0.017	-0.0096	0.41	-0.0096
	(0.023)	(0.023)	(0.023)	(0.023)	(1.2)	(0.27)	(0.23)	(0.27)
LHTL	0.026	0.027	0.035	0.035	0.024	0.026	0.79	0.26
	(0.025)	(0.025)	(0.025)	(0.025)	(1.3)	(0.29)	(0.27)	(0.29)
σ^2^	0.0011	0.0011	0.0011	0.0011	2.9	0.14	0.17	0.14
	(0.00042)	(0.00042)	(0.00043)	(0.00043)	(0.71)	(0.035)	(0.047)	(0.045)

## Discussion

In 2008, Barker and Schofield [7] suggested that “to correctly adopt the type of inference advocated by Batterham and Hopkins [6], sport scientists need to use fully Bayesian methods of analysis”. They also noted that most sport scientists are not trained in Bayesian methods, likely because this approach has only become commonplace as a statistical technique in approximately the last 20 years. To help make the Bayesian approach more accessible for those working in exercise science and sports medicine, we have provided here both a worked example (using statistical software) together with a description of the underlying models. We hope that this template will encourage those who deal with small samples and small effects to explore the full Bayesian method, which is well suited to the analysis of small samples. Other supporting information, where available, can be represented via the prior and hence formally and transparently incorporated with the data. In the absence of such information, the uncertainty induced by small samples is properly incorporated in the posterior estimates and inferences. In both of these situations, the analytical decision-making is enhanced, in support of the ultimate practical/clinical decision-making undertaken by sports practitioners.

### Case study re-interpreted with Bayesian inferences

An experimental study by Humberstone-Gough and colleagues reported changes (mean ± 90% confidence interval) in Hbmass of -1.4 ± 4.5% for IHE compared with Placebo and 3.2 ± 4.8% for LHTL compared with Placebo [9]. For RunEcon the authors reported ‘no beneficial changes’ for IHE compared with Placebo, and a change of 2.8 ± 4.4% for LHTL compared with Placebo. Although the analyses were undertaken using different outcome measures and a slightly different analytical model, the conclusions based on the posterior estimates and probabilities obtained from the Bayesian analysis reported above are broadly consistent with those reported by Humberstone-Gough *et al*. Importantly, the Bayesian approach allows a much more direct probabilistic interpretation of credible intervals and posterior probabilities; for example, the probability that the mean change in Hbmass after LHTL compared with the change after IHE is greater than the smallest worthwhile change (0.2) is 0.96.

Cohen’s effect size magnitudes are well established [11] but the selection of a small effect (*d = 0*.*2*) as the threshold value for a worthwhile change or difference has been questioned. In the sporting context, worthwhile changes in competition performance, which can alter medal rankings, have been derived [25] as approximately 0.3 times the within-subject standard deviation [26, 27], or ~0.3–1% of performance time in a range of sports [28–30]. Empirical evidence confirms that small effects (on competitive performance) are worthwhile for elite athletes and of practical relevance for coaches and scientists attempting to understand the likely benefit or harm of training regimen, lifestyle intervention or change in technique. The full Bayesian approach provides a robust and acceptable method of estimating the likelihood of a small effect. For instance, in the Humberstone-Gough et al. case study Hbmass increased ~21 g (or by 2.3%) more in LHTL vs IHE. Given that every gram of hemoglobin can carry ~4 mL O_2_, [31], it is reasonable to infer that this small increase in Hbmass is likely beneficial to overall oxygen transport capacity. The corresponding 95% credible interval for this comparison of absolute change ranged from -11.8 to +53.8 g, but on balance the probability is >0.8 that the true increase in Hbmass is substantial (worthwhile), which should be sufficient encouragement for most scientists and coaches to utilize altitude training to increase Hbmass–a position also supported by a meta-analysis of Hbmass and altitude training [23]. Likewise in the Humberstone-Gough et al. case study RunEcon improved (was lower) by ~0.17 L.min^-1^ (or lower by 4.2%) more in LHTL vs IHE. The associated 95% credible interval for this comparison of relative change ranged from –0.9 to -7.5%, with a probability of ~0.99 that the true decrease in submaximal oxygen consumption is substantial (worthwhile). Although contentious [32], an improved running economy after altitude training is advantageous to distance running performance because it reduces the utilization of oxygen at any given steady-state running speed [33, 34].

### Limitations of quasi-Bayesian approaches

Batterham and Hopkins (2006) have challenged the frequentist approach as being too conservative, and provided a useful, if somewhat unconventional, framework for interpreting small effects. The so-called magnitude-based approach emerging in sports science [18, 26, 35] is based on defining and justifying clinically, practically or mechanistically meaningful values of an effect. Confidence intervals are then used to interpret uncertainty in the effect in relation to these reference or threshold values. Much discussion has centred on the legitimacy of using vague priors in the magnitude-based approach and whether prior knowledge is actually useful in all cases [36]. There are inferential limitations to their approach [7, 8] which can be circumvented by using a full Bayesian approach that we have elaborated here.

A major criticism of the approach suggested by Batterham and Hopkins (2006) is that, contrary to the authors’ claims, their method is not (even approximately) Bayesian and that a Bayesian formulation of their approach would indeed make prior assumptions about the distribution of the true parameter values. Barker and Schofield (2008) suggest that the underlying prior distribution would be uniform, which makes a clear assumption about the parameter values (that any parameter value in the defined range is equally likely) and which can be influenced by transformations of the parameter. As demonstrated in our paper, a Bayesian formulation of the problem considered by Batterham and Hopkins (2006) can quite easily be constructed, using a reference prior which is arguably vague (often referred to as the Jeffreys prior [10]). Moreover, there are clear and natural links between the frequentist distributions based on sampling theory and the Bayesian posterior distributions under these prior assumptions. The use of the reference prior for the estimation and comparison problem considered in this paper is well-founded, theoretically sound and very commonly employed [10]. As discussed in the Methods section, however, other priors can also be considered, particularly if there is other information available to complement the analysis.

Another criticism levelled at Batterham and Hopkins (2006) by Barker and Schofield (2008) is their choice and use of an expanded set of categories, based on a non-standard choice of the thresholds used to define the categories, the use of different thresholds for different problems (e.g., sometimes 0.025 and 0.975 instead of 0.05 and 0.95), and the descriptors used to label the categories, namely ‘almost certainly not,…almost certainly’). However, while the expanded set of categories proposed by Batterham and Hopkins is not ‘standard’ in classical statistics, this does not mean that it is wrong, misleading or not useful. Indeed, such categorizations can be very useful *if* they are clearly justified, interpreted properly and provide additional decision support for clinical (or, in this case, sporting) interventions. Even in ‘traditional’ statistics, some statisticians suggest that a p-value less than 0.10 indicates ‘substantive’ evidence against the null hypothesis, while other statisticians would not counsel this. Similarly, although a p-value of 0.05 is almost overwhelmingly taken as the ‘significance level’, many statisticians strongly advise against its unconsidered use and suggest that other levels (such as 0.01 or 0.10) may be more appropriate for certain problems and desired inferences. A number of commentators in the sports science field have made similar observations [1, 37, 38]. The overwhelming advice is that the probabilities obtained as a result of statistical analysis must be useful in providing decision support for the problem at hand, and different probabilities can indeed be used if they are well justified, transparently reported and correctly interpreted.

The technical interpretation of a (frequentist) confidence interval is poorly understood by many practitioners. This has caused, and will continue to lead to, clumsy statements about the inferences that can be made on its basis. In contrast, an analogous Bayesian interval is directly interpretable: for example, a 95% credible interval indicates that the true parameter lies within this interval with an estimated probability of 0.95. Moreover, the analysis can be used to obtain other decision support statements such as a set of meaningful probabilities; for example, as demonstrated in the case study, one can obtain the probability that a particular parameter exceeds an objectively-derived threshold of clinical/practical/sporting interest. Of course, the particular decisions that are made on the basis of these probabilities remain the prerogative of the decision-maker. For example, the outcome of an intervention to improve athletic performance (e.g. a new experimental therapeutic treatment) may be classified as ‘possible’ in some cases (acceptable probability of improving performance, within minimal adverse effects, low cost, readily available, and legal in terms of anti-doping regulations), and hence lead to a decision of using, whereas in another context it may be deemed too risky (unacceptable risk of impairing performance, adverse effects on health and well-being, high cost and limited availability, and some uncertainty in meeting anti-doping regulations) and lead to no action. In practice, these decisions may not coincide with the traditional statement of a statistically significant effect at a 5% level [36]. In both cases, however, the decisions are enhanced by the richer probabilistic and inferential capability afforded by the Bayesian analysis.

In the context of small samples such as those encountered in this study, it is important to understand the nature and implications of the statistical assumptions underlying the adopted models and inferences. For example, in a standard linear regression model a common assumption is that the residuals (the differences between the observed and predicted values) are normally distributed. Note that this only applies to the residuals, not the explanatory or response variables. This assumption was also adopted in the model and analysis presented in this paper. There is a rich literature about the appropriateness of this assumption for small sample sizes. Importantly, if the residuals are indeed normally distributed then the regression estimates will possess all three desirable statistical characteristics of unbiasedness, consistency, and efficiency among all unbiased estimators; however, even if they are not normally distributed they will still be unbiased (accurate) and consistent (improve with increasing sample size) but will only be most efficient (i.e. have smallest variance) among a smaller class of (linear unbiased) estimators [39]. The most obvious implication of non-normal residuals is that the inferences may not be as sharp, but by virtue of the central limit theorem the sampling distribution of the coefficients will approach a normal distribution as the sample size increases, under mild conditions. In our study, this was achieved by employing a single residual term across all groups which effectively increased the sample size. Feasible alternatives would have been to allow different residual variances for each group or to employ a robust regression approach, for example using a *t* distribution for the errors. It is also noted that the Bayesian estimates avoid some of the inferential concerns, since the credible intervals and probabilistic rankings are obtained from the MCMC samples, i.e., from the posterior distributions themselves, as opposed to relying on stronger asymptotic assumptions that are required for frequentist inferences

Another topical issue that has substantive implications for small sample analysis is reproducibility [40]. Indeed, the very measure of reproducibility arguably faces similar challenges as those reported here, and a Bayesian approach is arguably preferable over measures based on p-values or confidence intervals [41–43]. See also a recent blog article that discusses this topic (http://alexanderetz.com/2015/08/30/the-bayesian-reproducibility-project/). The current debates are often conducted in the context of large samples, so the challenge is much greater for studies such as the one presented here. This is another topic for future research.

## Conclusion

We have demonstrated that a Bayesian analysis can be undertaken for small scale athlete studies and can yield comparable, but more directly interpretable and theoretically justified probabilistic outcomes compared with the so-called magnitude-based (quasi-Bayesian) approach. The model described here is one of the simplest Bayesian formulations, and can be expanded as needed to address other issues. Analytical approaches for small sample studies using full Bayesian, quasi-Bayesian, and frequentist decisions must be well justified, reported transparently and interpreted correctly.

## Supporting Information

S1 Table Data used in the case study. (DOCX)

S1 Text R code used in the analysis of the case study. (DOCX)
