# Supplementary material for: Bayesian Estimation of Small Effects in Exercise and Sports Science
Source: PLoS One. 2016 Apr 13;11(4):e0147311. doi: 10.1371/journal.pone.0147311 (PMC4830602; doi:10.1371/journal.pone.0147311)
Supplement: S1 Table — (DOCX) [file pone.0147311.s001.docx]

**S1 Table.** Data used in the case study

| **Person** | **Group** | **X** | **HbmassPre** | **HbmassPost** | **RunEconPre** | **RunEconPost** | **LamaxPre** | **LamaxPost** |
| --- | --- | --- | --- | --- | --- | --- | --- | --- |
|  |  |  | **(g)** | **(g)** | **(L/min)** | **(L/min)** | **(mmol/L)** | **(mmol/L)** |
| 1 | IHE | 6.176084 | 1052 | 1036 | 3.705 | 3.715 | 9.4 | 7.9 |
| 2 | IHE |  | 636 | 657 | 2.61 | 2.625 | 8.7 | 6.0 |
| 3 | IHE | 32.20081 | 604 | 603 | 2.21 | 2.105 | 8.4 | 6.3 |
| 4 | IHE | 7.548209 | 923 | 965 | 3.67 | 3.745 | 7.9 | 8.2 |
| 5 | IHE | 40.71281 | 890 | 900 | 3.685 | 3.775 | 11.6 | 7.3 |
| 6 | IHE | 51.10574 | 933 | 925 | 3.57 | 3.525 | 6.4 | 7.3 |
| 7 | IHE | 33.55932 | 886 | 892 | 3.72 | 3.605 | 7.7 | 8.0 |
| 8 | IHE | 28.1249 | 1099 | 1078 | 3.97 | 3.72 | 10.3 | 8.3 |
| 9 | LHTL | 9.045082 | 938 | 970 | 3.885 | 3.755 | 10.4 | 10.6 |
| 10 | LHTL | 0.293844 | 622 | 648 | NA | NA | 7.3 | 8.0 |
| 11 | LHTL | -16.9149 | 630 | 629 | 2.785 | 2.56 | NA | NA |
| 12 | LHTL | 60.48738 | 974 | 1028 | 3.82 | 3.565 | 10.1 | 6.8 |
| 13 | LHTL | 6.754492 | 889 | 952 | 3.75 | 3.6 | 12.4 | 9.3 |
| 14 | LHTL | 25.57932 | 1119 | 1148 | 4.375 | 4.27 | 8.1 | 6.7 |
| 15 | LHTL | 30.55809 | 912 | 924 | NA | NA | NA | NA |
| 16 | .Placebo | 76.73727 | 1063 | 1040 | 4.095 | 3.93 | 8.4 | 10.2 |
| 17 | .Placebo | 140.9874 | 959 | 1029 | 3.535 | 3.6 | 10.0 | 9.1 |
| 18 | .Placebo | 42.89953 | 615 | 613 | 2.27 | 2.205 | 10.9 | 10.8 |
| 19 | .Placebo | 57.97665 | 829 | 841 | 3.57 | 3.515 | 14.1 | 14.1 |
| 20 | .Placebo | 50.1382 | 820 | 869 | 3.49 | 3.375 | 7.0 | 8.2 |
| 21 | .Placebo | 109.9428 | 824 | 805 | NA | NA | 9.3 | 9.7 |
| 22 | .Placebo |  | 557 | 563 | NA | NA | NA | NA |
| 23 | .Placebo | 78.45364 | 532 | 554 | NA | NA | NA | NA |
