# Supplementary material for: Bayesian Estimation of Small Effects in Exercise and Sports Science
Source: PLoS One. 2016 Apr 13;11(4):e0147311. doi: 10.1371/journal.pone.0147311 (PMC4830602; doi:10.1371/journal.pone.0147311)
Supplement: S1 Text — (DOCX) [file pone.0147311.s002.docx]

**S1 Text.** R code used in the analysis of the case study

### ---------------------------------------------------

# 3 outcomes, univariate analysis:

# HMabs, REabs, RMLabs, HMrel, RErel and RMLrel

# NB: Choices in code below are indicated by #TODO#

### ---------------------------------------------------

# Setup

N=23 # no. participants

cburn=50000 # MCMC burnin

ckeep=100000 # MCMC iterations after burnin

citer=cburn+ckeep # total MCMC iterations

### --------

# Read data

cg=read.csv("Data.csv")

head(cg)

cg=cg[1:N,]

attach(cg)

### --------

## Make responses

# abs values

HMabs=HbmassPost-HbmassPre

REabs=RunEconPost-RunEconPre

RMLabs=LamaxPost-LamaxPre

# rel values

HMrel=(HbmassPost-HbmassPre)/HbmassPre

RErel=(RunEconPost-RunEconPre)/RunEconPre

RMLrel=(LamaxPost-LamaxPre)/LamaxPre

# 3D scatterplots

library(scatterplot3d)

scatterplot3d(HMabs, REabs, RMLabs, pch=16, highlight.3d=TRUE, type="h",xlab="Hbmass", ylab="RunEcon", zlab="La-max")

scatterplot3d(HMrel, RErel, RMLrel, pch=16, highlight.3d=TRUE, type="h", xlab="Hbmass", ylab="RunEcon", zlab="La-max")

### --------

## TO DO ##

# set responses for analyses: absolute (abs) or relative (rel) values

HM = HMabs

RE = REabs

RML = RMLabs

#HM = HMrel

#RE = RErel

#RML = RMLrel

### --------

## Exploratory plots

par(mfrow=c(2,2))

hist(X, main="", ylab="")

plot(X,HM,xlab="X", ylab="Hbmass")

plot(X,RE,xlab="X", ylab="RunEcon")

plot(X,RML,xlab="X", ylab="La-max")

par(mfrow=c(2,3))

stripchart(HM~Group,xlab="Hbmass")

stripchart(RE~Group,xlab="RunEcon")

stripchart(RML~Group,xlab="La-max")

plot(HM,RE, xlab="Hbmass", ylab="RunEcon")

plot(RML,RE,xlab="La-max", ylab="RunEcon")

plot(HM,RML,xlab="Hbmass", ylab="La-max")

par(mfrow=c(2,2))

stripchart(HaemoglobinMassPre~Group, xlab="Hbmass Pre")

stripchart(RunningEconomyPre~Group, xlab="RunEcon Pre")

stripchart(RunninMaxLactatePre~Group, xlab="La-max Pre")

### --------

## Analyses using MCMCpack

library(MCMCpack)

# make data lists

datHM=list(HM,X,Group)

datRE=list(RE,X,Group)

datRML=list(RML,X,Group)

# Uninformative priors

# b0, B0 are prior mean and precision for beta; B0=0 is equivalent to improper uniform prior

outHM <- MCMCregress(HM~X+as.factor(Group),b0=0,B0=0,data=datHM, marginal.likelihood="Chib95", burnin=cburn, mcmc=ckeep)

outRE <- MCMCregress(RE~X+as.factor(Group),b0=0,B0=0,data=datRE, marginal.likelihood="Chib95", burnin=cburn, mcmc=ckeep)

outRML <- MCMCregress(RML~X+as.factor(Group),b0=0,B0=0,data=datRML, marginal.likelihood="Chib95", burnin=cburn, mcmc=ckeep)

summary(outHM)

summary(outRE)

summary(outRML)

# Informative priors - ONLY FOR HM

# Meta-analysis + Gore pers comm

# b0, B0 are prior mean and precision for beta

#gb0 = c(0,0.0, 0.0, 2.6)

#gB0 = diag(c(1,1,5, 5))

#outHM <- MCMCregress(HM~X+as.factor(Group),b0=gb0,B0=gB0,data=datHM, marginal.likelihood="Chib95", burnin=cburn, mcmc=ckeep)

#summary(outHM)

# calculate LHTL - IHE effects

diffHM=outHM[,4]-outHM[,3]

diffRE=outRE[,4]-outRE[,3]

diffRML=outRML[,4]-outRML[,3]

# plot posterior densities

par(mfrow=c(3,1))

plot(density(diffHM),xlab="",ylab="", main="Hbmass")

#main="Hbmass", sub="LHTL-IHE (solid), LHTL-Placebo (dotted), IHE-Placebo (dashed)",

#ylim=c(0,25), xlim=c(-0.10, 0.10))

lines(density(outHM[,3]),lty=2) # IHE - Placebo

lines(density(outHM[,4]),lty=3) # LHTL - Placebo

plot(density(diffRE),xlab="", ylab="", main="RunEcon")

#main="RunEcon", sub="LHTL-IHE (solid), LHTL-Placebo (dotted), IHE-Placebo (dashed)",

#ylim=c(0,25), xlim=c(-0.10, 0.10))

lines(density(outRE[,3]),lty=2) # IHE - Placebo

lines(density(outRE[,4]),lty=3) # LHTL - Placebo

plot(density(diffRML),xlab="", ylab="", main="La-max")

#main="La-max", sub="LHTL-IHE (solid), LHTL-Placebo (dotted), IHE-Placebo (dashed)",

#ylim=c(0,25), xlim=c(-0.10, 0.10))

lines(density(outRML[,3]),lty=2) # IHE - Placebo

lines(density(outRML[,4]),lty=3) # LHTL - Placebo

# Posterior estimates

## --------------

## TO DO ##

# choose output

## options outHM and diffHM, outRE and diffRE, outRML and diffRML

outt = outHM

difft = diffHM

## -------------

# summary X, IHE, LHTL, LHTL-IHE: mean,sd, 95%CI, 90%CI

c(mean(outt[,2]),sd(outt[,2]),quantile(outt[,2],c(0.025, 0.975)),quantile(outt[,2],c(0.05, 0.95)))

c(mean(outt[,3]),sd(outt[,3]),quantile(outt[,3],c(0.025, 0.975)),quantile(outt[,3],c(0.05, 0.95)))

c(mean(outt[,4]),sd(outt[,4]),quantile(outt[,4],c(0.025, 0.975)),quantile(outt[,4],c(0.05, 0.95)))

c(mean(difft),sd(difft),quantile(difft,c(0.025, 0.975)),quantile(difft,c(0.05, 0.95)))

# cohens D

IvP = outt[,3]/sd(outt[,3])

LvP = outt[,4]/sd(outt[,4])

LvI = difft/sd(difft)

# summaries Cohens D: mean, sd, 95%CI, 90% CI

# IHE vs Placebo

c(mean(IvP),sd(IvP),quantile(IvP,c(0.025, 0.975)),quantile(IvP,c(0.05, 0.95)))

# LHTL vs Placebo

c(mean(LvP),sd(LvP),quantile(LvP,c(0.025, 0.975)),quantile(LvP,c(0.05, 0.95)))

# LHTL vs IHE

c(mean(LvI),sd(LvI),quantile(LvI,c(0.025, 0.975)),quantile(LvI,c(0.05, 0.95)))

# Prob. Cohen's D <> 0.2

# IHE vs Placebo

c(mean(IvP < -0.2), mean(IvP > 0.2))

# LHTL vs Placebo

c(mean(LvP < -0.2), mean(LvP > 0.2))

# LHTL vs IHE

c(mean(LvI < -0.2), mean(LvI > 0.2))

# predicted perfomance on each treatment

nr=ckeep

yeP = matrix(0,nrow=nr, ncol=N)

yeI = matrix(0,nrow=nr, ncol=N)

yeL = matrix(0,nrow=nr, ncol=N)

ypP = matrix(0,nrow=nr, ncol=N)

ypI = matrix(0,nrow=nr, ncol=N)

ypL = matrix(0,nrow=nr, ncol=N)

ymP = matrix(0,nrow=k, ncol=3)

ymI = matrix(0,nrow=k, ncol=3)

ymL = matrix(0,nrow=k, ncol=3)

yrP = matrix(0,nrow=nr, ncol=N)

yrI = matrix(0,nrow=nr, ncol=N)

yrL = matrix(0,nrow=nr, ncol=N)

yrPmean = matrix(0,nrow=N, ncol=5)

yrImean = matrix(0,nrow=N, ncol=5)

yrLmean = matrix(0,nrow=N, ncol=5)

par(mfrow=c(1,1))

for (k in 1:N){ # expected performance

yeP[,k]=outt[,1] + outt[,2]*X[k]

yeI[,k]=outt[,1] + outt[,2]*X[k] + outt[,3]

yeL[,k]=outt[,1] + outt[,2]*X[k] + outt[,4]}

for (k in 1:N){ # predicted performance

ypP[,k]=outt[,1] + outt[,2]*X[k] + rnorm(nr,0,sqrt(outt[,5]))

ypI[,k]=outt[,1] + outt[,2]*X[k] + outt[,3] + rnorm(nr,0,sqrt(outt[,5]))

ypL[,k]=outt[,1] + outt[,2]*X[k] + outt[,4]} + rnorm(nr,0,sqrt(outt[,5]))

# plot boxplot of expected and predicted performance for I # Save separately eg PredPerfIHE + ResponseName(HMrel, RMLrel)

boxplot(yeI[,1:N],range=0,xlab="Subject",ylab="Predicted", sub="Expected performance of each subject on IHE")

boxplot(yeL[,1:N],range=0,xlab="Subject",ylab="Predicted", sub="Expected performance of each subject on LHTL")

boxplot(ypI[,1:N],range=0,xlab="Subject",ylab="Predicted", sub="Predicted performance of each subject on IHE")

boxplot(ypL[,1:N],range=0,xlab="Subject",ylab="Predicted", sub="Predicted performance of each subject on LHTL")

# plot scatterplot of expected and predicted performance for IHE versus LHTL # Save separately eg PredPerfIHELHTL + ResponseName(HMrel, RMLrel)

plot(yeI[,1:N],yeL[,1:N],xlab="IHE", ylab="LHTL", sub="Expected performance")

abline(0,1)

plot(ypI[,1:N],ypL[,1:N],xlab="IHE", ylab="LHTL", sub="Predicted performance")

abline(0,1)

# mean predictive performance - comparison of IHE and LHTL # same as MeanPredPerfIHTLHTL + Response

for (k in 1:N){

ymP[k,] = c(mean(ypP[,k]), quantile(ypP[,k],c(0.025, 0.975),na.rm=T))

ymI[k,] = c(mean(ypI[,k]), quantile(ypI[,k],c(0.025, 0.975),na.rm=T))

ymL[k,] = c(mean(ypL[,k]), quantile(ypL[,k],c(0.025, 0.975),na.rm=T))}

plot(ymI[,1],ymL[,1],type="p", xlab="Predicted IHE", ylab="Predicted LHTL", sub="Mean predicted performance of subjects on IHE vs LHTL",)

abline(0,1)

# ranks of subjects based on expected performance # save as RankPerfIHE + Response etc

# note: for the rel analyses this just follows the ranks of X since the effects of treatment are so small

for (k in 1:nr){

yrP[k,] = rank(yeP[k,], na.last="keep")

yrI[k,] = rank(yeI[k,], na.last="keep")

yrL[k,] = rank(yeL[k,], na.last="keep")}

boxplot(yrI[,1:N],range=0,xlab="Subject",ylab="Rank", sub="Rank of each subject on IHE")

boxplot(yrL[,1:N],range=0,xlab="Subject",ylab="Rank", sub="Rank of each subject on LHTL")

# quantiles of the ranks for each subject (0.10, 0.25, 0.5, 0.75, 0.90)

for (k in 1:N){

yrImean[k,] = c(quantile(yrI[,k],c(0.10, 0.25, 0.5, 0.75, 0.90),na.rm=TRUE))

yrLmean[k,] = c(quantile(yrL[,k],c(0.10, 0.25, 0.5, 0.75, 0.90),na.rm=TRUE))}

yrImean

yrLmean

## End of code
